# Effects of Quartz Sand on the Electromagnetic Wave Absorption of Cementitious Materials

**DOI:** 10.3390/ma17235795

**Published:** 2024-11-26

**Authors:** Chaoqun Li, Zixiao Wang, Weizheng Shi, Ling Huang, Aming Xie

**Affiliations:** 1 School of Safety Science and Engineering, Nanjing University of Science and Technology, Nanjing 210094, China; 2 School of Mechanical Engineering, Nanjing University of Science and Technology, Nanjing 210094, China

**Keywords:** mortar, electromagnetic absorption, thickness, cement-to-sand ratios, water-to-cement ratios

## Abstract

The roles of dielectric materials in adjusting the electromagnetic wave (EMW) absorption performance of an EMW absorber are as crucial as the EMW absorbents. The commonly used cement-based materials, such as mortar, are typical composites of multiple dielectric materials, such as quartz sand and air in the pores. This study investigates the EMW-absorption performances within the frequency range of 2 GHz to 18 GHz of cement paste and mortar samples with different sand-to-cement ratios (S/C), water-to-cement ratios (W/C), and thicknesses. The bow-frame method is used to measure the EMW reflection loss (RL) curves of slab-like samples. The coaxial method is used to record the electromagnetic parameters of the sample powders, which are also used to calculate the EMW RL curves. The results prove that the EMW-absorption performances of a slab-like mortar sample are monotonically related to the S/C ratio and the actual air volume, which is closely related to the thickness.

## 1. Introduction

Electromagnetic radiation is a common form of radiation in the atmosphere. With the rapid development of wireless communication systems, high-frequency electrical products have provided great convenience for people’s health but also made the urban electromagnetic fields more and more complex. Electromagnetic radiation in urban cities is becoming increasingly severe [1,2,3], and has become the fourth most significant source of pollution in the world [4], and is hazardous to human health, electronic equipment [5,6,7], and military stealth applications [8,9,10]. Cementitious materials are the commonly used building materials in military protection buildings and infrastructure constructions, and they have excellent mechanical properties, durability, and affordability [11]. Some building materials have varying degrees of potential to absorb EMWs (electromagnetic waves), such as conductive fiber-reinforced concrete [12,13,14], ferrocene-based geopolymers [15,16], and gypsum composites [17,18]. To better reduce the harm caused by electromagnetic radiation, researchers are committed to designing building materials that absorb EMWs.

The EMW transmission properties of cementitious materials are affected by many factors, including the combined effect of the cementitious matrix, dielectric phases and the electromagnetic phases, dielectric constant [19], magnetic permeability [20], and pore structure [21,22]. Good impedance-matching properties are also crucial for materials to achieve efficient EMW-absorption properties [23]. The larger the mixing ratio of the dielectric phases with a low dielectric constant to cement, the lower the overall dielectric constant of the composite and the better the EMW transmission properties [24]. Therefore, adding some dielectric materials, such as expandable polystyrene [25,26], polyvinyl alcohol, SiO_2_ fibers, hollow glass microspheres [27], and glass powders [28], to cementitious materials can improve the impedance-matching properties of composites, resulting in better EMW transmission and absorption properties. 

Quartz sand, a typical dielectric material with a low dielectric constant, like air, is a critical component of modern concrete. In general, sand is always used to improve the strength of hardened concrete. However, the wave absorption properties of mortar reported in the literature are poor [13,29,30,31,32], and the effective bandwidth values of slab-like mortar samples are smaller than 5 GHz, as shown in Table 1. In theory, quartz sand can regulate the dielectric properties of cementitious composites, which may improve the impedance-matching results of the composites and enhance the absorption of EMWs. However, the sand-to-cement ratios in regular mortars are relatively fixed, which may cause an impedance mismatch in the matrix and result in poor EMW absorption. The impedance-matching behaviours of cement mortar samples should be considered as the combined effects of quartz sand, the pore structure in the hardened slurry, and cement hydration products. 

In this work, the influences of quartz sand and air in pores on the EMW-absorption performance of cementitious materials are investigated. The contents of quartz sand and the air in the mortar matrix are adjusted by changing the water-to-cement ratios (W/C) and the thickness of the samples. The absorption polarity properties of mortar samples are analyzed by determining electromagnetic parameters. The results of the study will provide new strategies for the development of high-performance electromagnetic absorption cementitious materials.

## 2. Experimental

### 2.1. Materials and Sample Preparation

**Figure 1:**
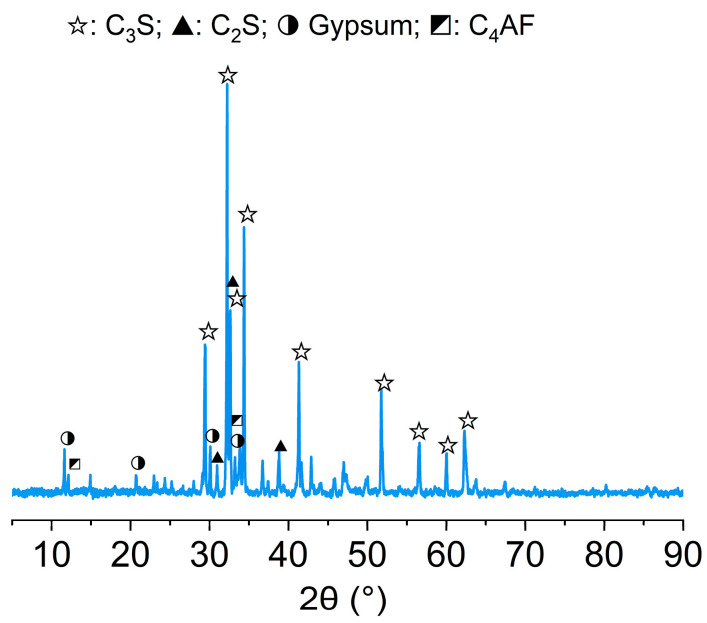
XRD pattern of cement powders.

**Figure 2:**
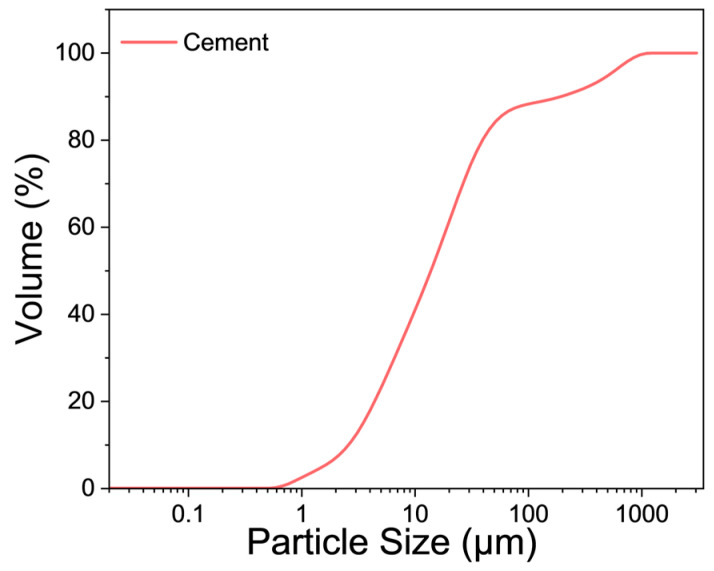
Particle size distribution of cement powders.

The P.II 42.5 cement powders and ISO standard quartz sand were used to prepare samples. The chemical composition of cement was measured by X-ray fluorescence (Axios, Malvern Panalytical Ltd., Malvern, UK), and the results are listed in Table 2. The X-ray powder diffraction (SmartLab9, Rigaku, Tokyo, Japan) spectrum of the cement powders is displayed in Figure 1. The particle size distribution curve of cement powders (Figure 2) was tested by a laser particle analyzer (Mastersize 3000, Malvern Panalytical Ltd., Malvern, UK). In order to regulate the state of the cement paste, an appropriate amount of hydroxypropyl methylcellulose (HPMC) was added during the test. After dry mixing cement, quartz sand, and HPMC powders, the mixed water was added to the mortar mixer to mix for another 5 min. The slurry was cast into samples of three different sizes (200 × 200 × 10 mm^3^, 200 × 200 × 20 mm^3^, and 200 × 200 × 30 mm^3^). Samples were de-molded after 24 h and cured for 28 d in a curing chamber at a temperature of 20 ± 2 °C and relative humidity higher than 95%. Mortar samples with five water-to-cement mass ratios (W/C), four sand-to-cement mass ratios (S/C), and three thicknesses were designed, and the mix proportions of samples in each group are shown in Table 3.

### 2.2. Methods

#### 2.2.1. EMWs Reflection Loss (RL) Performance

**Figure 3:**
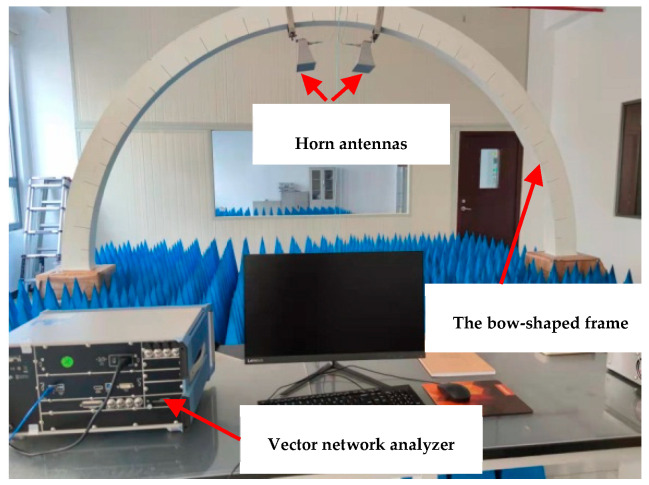
The measuring system of RL of slab-like samples by the bow-frame method.

The absorption properties of cementitious composites for EMWs are usually evaluated by RL values [33]. A vector network analyzer (ZNB43, Rohde&Schwarz, Columbia, MD, USA) was used to test the absorption performance of EMWs from 2 GHz to 18 GHz using the bow-frame method [27,34]. The measuring system of RL curves of slab-like samples is shown in Figure 3. It is important to ensure that all samples are dried at 55 °C for 48 h in order to remove the impact of free water on the results of the wave-absorbing property test [35,36]. 

The transmitter and receiver antennas of the test system are mounted on a segment of a circular frame, with the center of the sample plate coinciding with the center of the circle of the bowed frame. The vector network analyzer was calibrated prior to the test, after which the samples were positioned on a metallic reflector plate. The area surrounding the reflector plate was lined with pyramid-shaped absorptive foam to minimize interference. The EMW signals produced by the equipment were emitted via one horn antenna and captured by the other horn antenna. All data represent the average of three test results for each sample. The system was intended to measure the reflected power from a reflective metal plate (*P_m_*) and the sample (*P_a_*). The reflective loss of the test sample was calculated by the formula [36]:

(1) $$RL(dB)=10\mathrm{lg}\frac{P_{m}}{P_{a}}$$

The EMW-absorption characteristics are studied through the measurement of RL curves. Based on the reflection principle [37], if the values of RL curves are smaller than −10 dB, more than 90% of the electromagnetic energy is effectively absorbed. In this study, the frequency range where the RL value is below −10 dB was designated as the effective bandwidth (B_e_) for the sample being tested.

#### 2.2.2. Measurement and Simulation of Electromagnetic Parameters

(1) Measurement

The electromagnetic parameters (complex permittivity and complex permeability) of mortar samples were measured using the coaxial transmission method with the vector network analyzer (N5222B, Keysight Technologies, Santa Rosa, CA, USA). The samples were ground into powder and dried at 50 °C for 24 h. The sample powders were mixed with paraffin wax at a mass ratio of 9:1. The mixture was pressed into a mold with an inner diameter of 3.04 mm and an outer diameter of 7.00 mm to complete the sample making.

(2) Simulation of RL curves

According to the transmission line theory [38,39,40], the RL value was calculated using the following equation:

(2) $$RL(dB)=20\mathrm{lg}\left|\frac{Z_{in}-Z_{0}}{Z_{in}+Z_{0}}\right|$$

(3) $$Z_{in}=Z_{0}\sqrt{\frac{\mu^{\prime }-j\mu^{\prime \prime }}{\varepsilon^{\prime }-j\varepsilon^{\prime \prime }}}\tanh\left[j\left(\frac{2\pi ft}{c}\right)\sqrt{\left(\mu \prime -j\mu \prime \prime )(\varepsilon \prime -j\varepsilon \prime \prime \right)}\right]$$

(4) $$Z_{0}=\sqrt{\frac{\mu_{0}}{\varepsilon_{0}}}$$

where *Z_in_* and *Z*_0_ denote the input impedance at the surfaces of samples and the impedance in free space, respectively; *f*, *t*, and *c* stand for the frequency of EMWs, the thickness of the material, and the speed of light, respectively. *ε*′ and *μ*′ represent the real parts of permittivity and permeability, while *ε*″ and *μ*″ represent their imaginary parts. Lastly, *ε_0_* and *μ*_0_ correspond to the permittivity and permeability of free space. Meanwhile, Equations (3) and (4) indicate that the impedance of the absorbing material should closely match that of free space to maximize the absorption of incident EMWs. This implies that the permittivity and permeability of the material should be similar to those of free space. Consequently, the minimum RL is achieved when the input impedance (*Z_in_*) is nearly equal to the impedance of free space (*Z*_0_). For a perfect microwave absorber, RL should be infinitesimal [41]. Reflection loss can be influenced by numerous factors, such as impedance matching [25,42], the electromagnetic and physical properties of the absorber [43,44,45], the thickness and structural configuration of the samples [46,47,48], and the frequency of the EMWs [49]. For EMWs to be absorbed, they must first enter the samples.

As indicated in Equations (3) and (4), the electromagnetic properties of the material also influence the absorption of EMWs [50,51]. The dielectric constant reflects the ability to polarize in response to an electric field of a material, which includes two components: the real part (*ε*′) and the imaginary part (*ε*″). The tangent of the loss angle (*tan δ_ε_*) is determined using Equation (5).

(5) $$\tan\delta_{\varepsilon }=\frac{\varepsilon \prime \prime }{\varepsilon \prime }$$

where *tan δ_ε_* represents the tangent of the loss angle, indicating the capability of a material to dissipate electromagnetic energy during the polarization process. *ε*′ denotes the real component of the dielectric permittivity, which reflects the ability to store and release electromagnetic energy of a material. The *ε*″ signifies the imaginary part of the dielectric constant, reflecting the ability to dissipate electromagnetic energy of a material. A higher *ε*′ suggests poorer impedance matching between free space and the material surface. Increased values of *ε*″ and *tan δ_ε_* indicate greater dissipation of electromagnetic energy by the material [52].

The measured parameters of magnetic permeability include the real part (*μ*′) and the imaginary part (*μ*″). The magnetic loss angle tangent (*tan δ_μ_*) is determined using Equation (6).

(6) $$\tan\delta_{\mu }=\frac{\mu \prime \prime }{\mu \prime }$$

where *tan δ_μ_* denotes the tangent of the magnetic loss angle, *μ*′ signifies the real part of magnetic permeability, which reflects the magnetization level of the material; *μ*″ represents the imaginary part of magnetic permeability, indicating the electromagnetic loss due to the creation and reorientation of magnetic dipoles of a material. A lower *μ*″ and *tan δ_μ_* imply reduced magnetic and eddy current losses in the material. Conversely, higher values of *μ*′, *μ*″, and *tan δ_μ_* correlate with enhanced EMW-absorption properties of the material.

#### 2.2.3. Oven-Dry Porosity

At an age of 28 days, the samples were submerged in water at (20 ± 2) °C for 24 h. The water surface should be higher than the top surface of the sample for 25 mm. The water on the surfaces of the samples was wiped off with a wet towel, and then the masses of the samples were weighed. After that, the samples were dried in an oven at (50 ± 5) °C for 24 h. Again, the samples are weighed. The sample oven-dry porosity can be calculated in accordance with the following formula:

(7) $$P_{0}=\frac{m_{1}-m_{0}}{\rho_{w}V_{0}}\times 100(\%)$$

where *m*_1_ represents the mass of the sample after immersing in water for 24 h; *m*_0_ represents the mass of the sample after drying; *V*_0_ represents the volume of the sample; and *ρ_w_* represents the density of water.

## 3. Results

### 3.1. Electromagnetic Reflection Loss

#### 3.1.1. Effects of Sand-to-Cement Ratios

**Figure 4:**
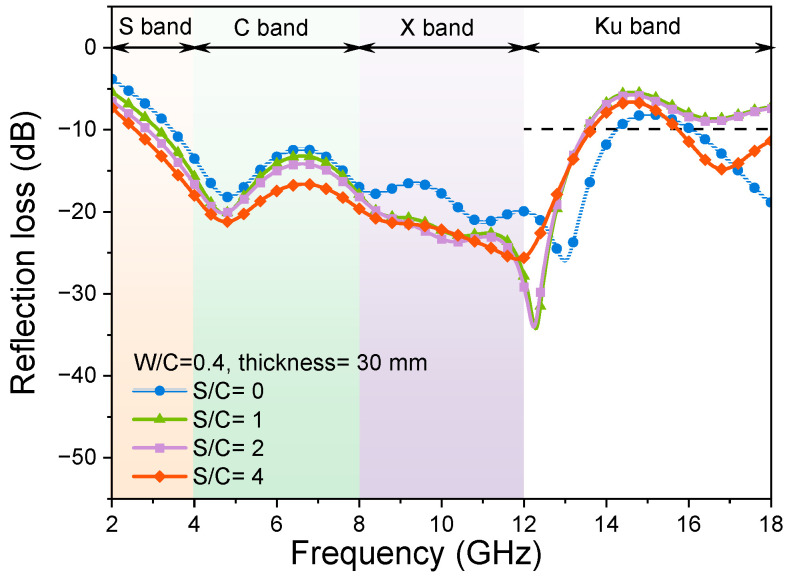
RL curves of samples with different S/C ratios and the thickness of 30 mm and W/C of 0.4.

**Figure 5:**
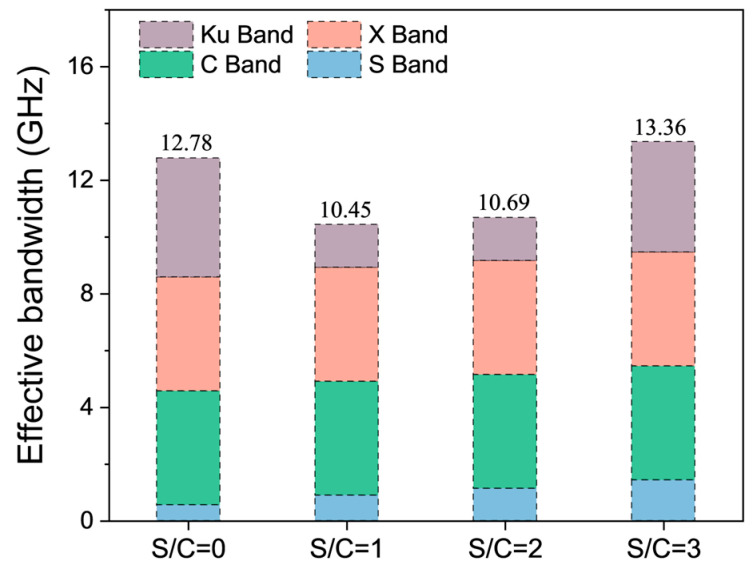
Effective bandwidth (B_e_) of samples with different S/C ratios and the same W/C of 0.4 and thickness of 30 mm.

Figure 4 shows the effects of variation of sand-to-cement ratios (S/C) on the EMW-absorption properties of cementitious composites in the range of 2 GHz to 18 GHz. In the RL curves, the more negative the value of RL, the stronger the absorption of EMWs. If the value of RL is below −10 dB, the width of the absorption band is referred to as the adequate absorption bandwidth when 90% of the energy of the EMWs is absorbed. The effective absorption bandwidths of samples with different S/C ratios are shown in Figure 5.

As shown in Figure 4, when the thickness is 30 mm, the samples with different S/C ratios show a relatively wide bandwidth of the EMWs. It can also be observed that all the samples can realize the effective absorption of the C band (4 GHz to 8 GHz) and X band (8 GHz to 12 GHz) EMWs, and the absorption of the X band and Ku band (12 GHz to 18 GHz) EMWs is slightly insufficient. From Figure 4, it can be observed that when the quartz sand content is lower (S/C = 1, 2), the RL curves have an upward trend in the S band (2 GHz to 4 GHz) and C band, a downward trend in the X band, and an upward trend in the Ku band compared with that of the blank sample (without sand). When the addition of quartz sand in the mortar samples is relatively high (S/C = 3), the absorption effect of the samples on the EMWs from 2 GHz to 12 GHz is enhanced, but the absorption of the samples on the EMWs from 12 to 18 GHz is much weakened. That is, the addition of quartz sand reduces the EMW absorption of the mortar samples in the low-frequency band. A larger S/C ratio can enhance the EMW absorption of mortar in the low-frequency band and weaken that in the high-frequency band. 

Before the addition of quartz sand, the hardened cement paste sample had one RL peak at 4.82 GHz and 12 GHz, with peak values of −18.2 dB and −25.97 dB, respectively. After the addition of quartz sand, there were also two RL peaks in the RL curves of mortar samples near 5 GHz and 12 GHz when S/C = 1 and 2, respectively. The results of EMW absorption of mortar samples shown in Figure 4 are pretty good compared with the data in the literature. For example, in reference [30], the RL values of the mortar sample are about −8 dB in the EMW frequency ranges of 1 GHz to 6 GHz. In reference [53], the B_e_ of the mortar sample is only 1.54 GHz within the EMW frequency of 2 GHz to 18 GHz, and the minimum RL value is −14.47 dB. However, it is worth noting that when the S/C ratio of the mortar sample is three, there is another RL peak at 10.46 GHz with a peak value of −29.69 dB. Therefore, the addition of quartz sand not only affects the values of peaks in the RL curves of samples, but also affects the number of RL peaks.

As can be seen from Figure 5, for the sample with a thickness of 30 mm, when the cement–sand ratio is increased from 0 to 3, the effective absorption bandwidth of the sample is above 10 GHz, and all of them can realize the full-band effective absorption of the C band and the X band. The absorption of samples with the S/C ratios of 1 and 2 on the Ku band EMWs is not very satisfactory. Meanwhile, the absorption of samples with S/C ratios of 0 and 3 on Ku band EMWs is improved. The effective absorption bandwidth of the sample with the S/C ratio of 3 is the largest, at 13.36 GHz. The effective absorption bandwidth values of the samples with the S/C ratios of 1 and 2 are similar, which are 10.45 GHz and 10.69 GHz, respectively. The effective absorption bandwidth of the sample without sand is 12.78 GHz. From the RL curves, this difference is mainly due to the absorption of EMWs by samples in the high-frequency band of 16 GHz to 18 GHz. As mentioned in the previous section, the addition of quartz sand weakens the absorption of the mortar sample of the EMWs with a high-frequency band.

#### 3.1.2. Effects of the Thickness of the Samples

**Figure 6:**
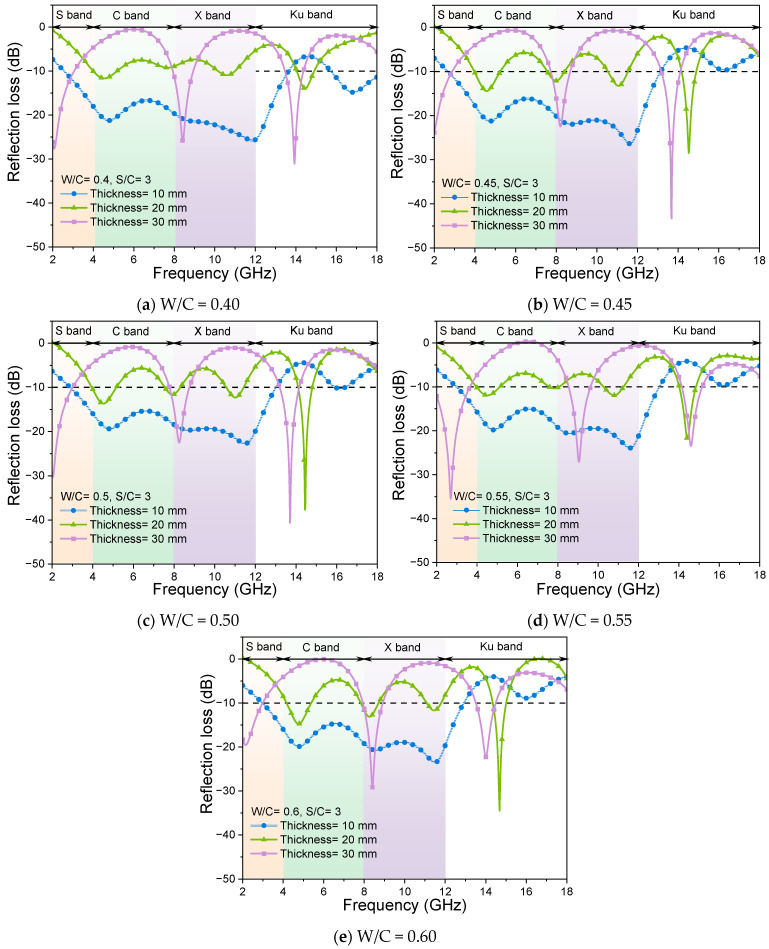
RL curves of mortar samples with different W/C ratios and thicknesses, and the same S/C ratio of 3.

**Figure 7:**
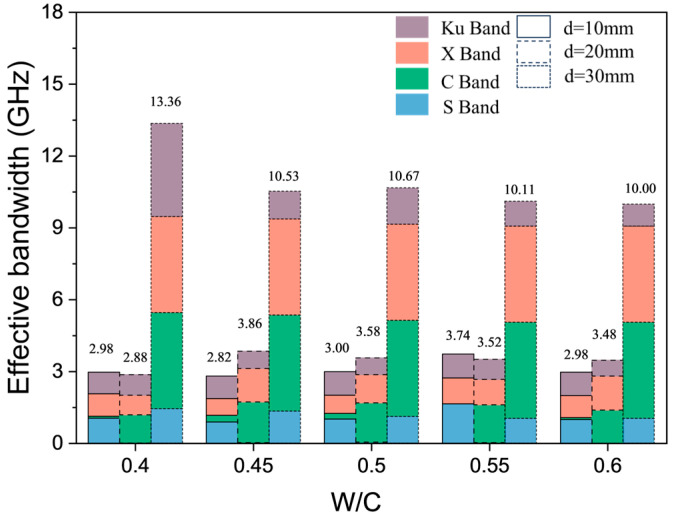
Effective bandwidth (B_e_) of samples with different W/C ratios and thicknesses, and the same S/C ratio of 3.

Changes in sample thickness also alter the actual quantity of quartz sand and the propagation distance of EMWs within the mortar, thereby affecting the scattering and RL curves. Figure 6 shows the effects of the thicknesses on the RL curves for the sample with the same sand-to-cement ratio. Figure 7 shows the effective absorption bandwidth values of all samples for this set of tests.

The impact of varying thickness on the wave absorption properties of cementitious composites with a water-to-cement (W/C) ratio of 0.40 is illustrated in Figure 6a. The peak of the curve of the sample with a thickness of 10 mm is −31 dB, but the effective absorption bandwidth is only 2.96 GHz (Figure 6). The appearance of absorption peaks can be attributed to the fact that each microwave-absorbing component exhibits specific frequency selectivity and possesses inherent absorption peaks within the wider frequency range. When the thickness of the sample is 20 mm or 30 mm, the RL curves of the sample tend to be flat, and start to change abruptly at 13 GHz and 12 GHz. Compared to the sample with a thickness of 10 mm, the mortar sample with a thickness of 20 mm shows a reduced peak RL curve and has barely any effective bandwidth. The RL curve of the sample with a thickness of 30 mm decreases compared with that of the sample with a thickness of 20 mm. The RL values are first enhanced and then decreased with the change in frequency; the peak value of RL curves can reach −25.78 dB and the effective absorption bandwidth is 13.3 GHz (Figure 7). As shown in Figure 6b–e, the mortar samples with higher W/C ratios present similar EMW-absorption properties to the mortar with the W/C ratio of 0.4. That is, the effects of thickness variation on the sample EMW RL curves seem not to be affected by the variation in W/C ratios of mortar samples. Thus, when designing absorbing cement-based materials, one can adjust the effective absorption band by altering the thickness of the samples.

The reason for the flattened EMW RL curves as the thickness of the mortar sample increases needs to be noted. Coherent loss occurs when two EMWs with the same frequency and a specific wave path difference are the main reason for the formation of the peaks of the EMW RL curves of the tested sample. When the reflectance of the absorber is measured using the bow-frame method, the EMWs can penetrate the thinner sample to form a secondary reflection wave, providing interference loss. With the increase in sample thickness, EMWs cannot penetrate the matrix effectively, resulting in an interference loss. When the thickness of the mortar sample is 30 mm, the EMWs in the frequency range of 2 GHz to 12 GHz are effectively absorbed, but the absorption capacity of EMWs in the frequency range of 12 GHz to 18 GHz sharply decreases. This is mainly because the low-frequency EMWs have a more robust penetration performance than the high-frequency EMWs and can enter into the sample, resulting in a stronger interference loss. In the frequency range of 12 GHz to 18 GHz, the interference loss is much higher than that of high-frequency EMWs, meaning EMWs are reflected on the sample surface.

#### 3.1.3. Effects of Water-to-Cement Ratios

**Figure 8:**
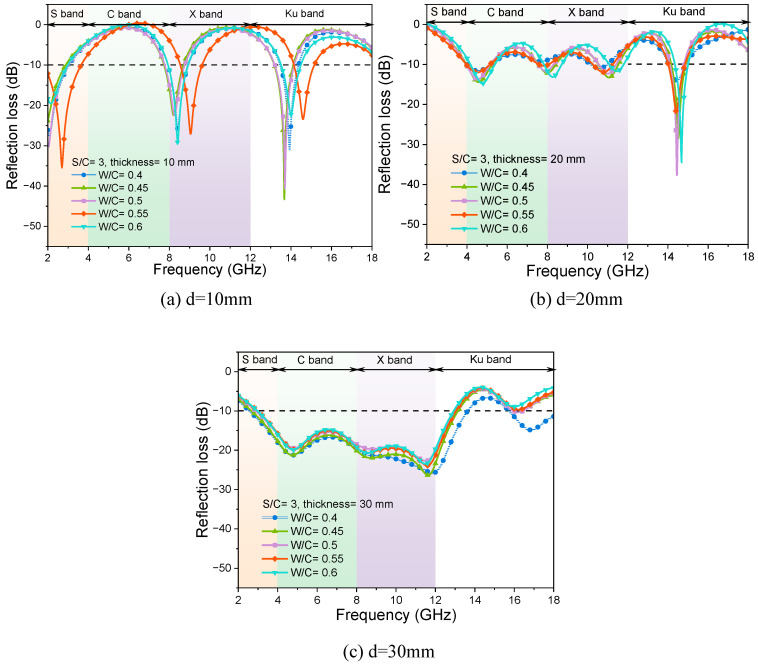
RL curves of mortar samples with different W/C ratios and thicknesses, and the same S/C ratio of 3.

In general, an increase in the water-to-cement ratios (W/C) of cement-based materials tends to elevate both their dielectric constant and electrical conductivity. The influence of the change in W/C ratios on the EMW-absorption performance of mortar test blocks with different thicknesses is shown in Figure 8. The electromagnetic RL curves of mortar samples with the same W/C and different thicknesses are similar for the absorption performance of EMWs in the frequency band from 2 GHz to 18 GHz. For mortar specimens with the same thickness, the change in W/C ratios has little effect on the EMW-absorption performance of mortar samples. The change in sand contents directly affects the dielectric constant and magnetic permeability of the cementitious materials. At the same time, the addition of quartz sand also affects the microstructure of the test matrixes and the reflection and scattering process of EMWs in the cementing materials. When the S/C ratio is fixed, the change in W/C ratios mainly affects the hydration process of cement and the microstructure in the hardened sample, but its influence on the EMW-adsorption property of the hardened mortar sample is limited.

It is worth noting that the RL curve of the mortar sample with a thickness of 10 mm and a W/C of 0.55 is significantly changed. This may be because the changes in pore structure and hydration products caused by the W/C ratio have obvious effects on the frequency selectivity of EMWs at the thickness of 10 mm. When the thickness of the test sample is 30 mm, the EMW RL curve of the mortar sample with W/C of 0.40 shifts down in the Ku band, and the peak value of the RL appears at 16.8 GHz with a value of −14.8 dB, which indicates that the sample also has a certain absorption ability to EMWs in the Ku band.

### 3.2. Electromagnetic Parameters

#### 3.2.1. Samples with Different Sand-to-Cement Ratios

**Figure 9:**
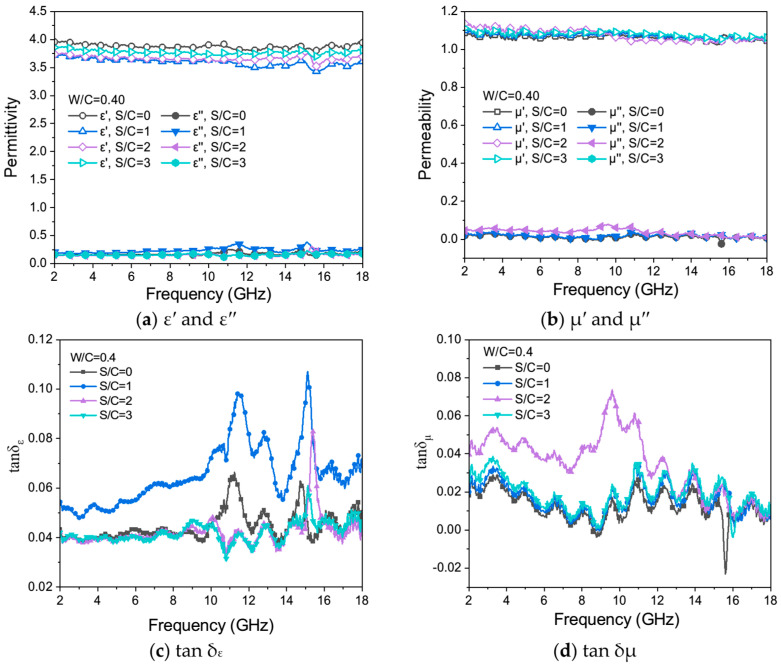
*ε*′ and *ε*″ (**a**), *μ*′ and *μ*″ (**b**), *tan δ_ε_* (**c**), and *tan δ_μ_* (**d**) of mortar samples with different S/C ratios and the same W/C of 0.4.

Figure 9a–d shows the variation of *ε*′ and *ε*″ (Figure 9a), *μ*′ and *μ*″ (Figure 9b), *tan δ_ε_* (Figure 9c), and *tan δ_μ_* (Figure 9d) for samples with different sand-to-cement ratios in the EMW ranges of 2 GHz to 18 GHz. 

In Figure 9a, all *ε*′ curves tend to be straight lines, and their values fluctuate between 3.5 and 4.0. The largest value of *ε*′ close to 4.0 was found for the sample without sand, and the smallest value of *ε*′ was found for the mortar sample with the S/C = 1. There is a slight peak appearing at an EMW frequency of 15 GHz, and the value of *ε*′ decreases first and then increases with the increase in the content of quartz sand in mortar samples, which suggests that the incorporation of quartz sand enhances the dielectric constants of the samples but the improvement is limited. All *ε*″ curves fluctuate around 0.2, and there is no obvious rule of change with the change in frequency. But the *ε*″ curves of the samples with S/C= 0 and 1 show two gentle peaks at EMW frequencies of 12 GHz and 15 GHz. This implies that cementitious materials possess specific polarization capabilities in an electromagnetic field; however, the electromagnetic losses resulting from the reorientation of electric dipoles remain relatively limited. The curves of *tan δ_ε_* of the sample with S/C = 0 and 1 are relatively flat in the EMW frequency range of 2 GHz to 10 GHz, as shown in Figure 9c, with only two slight peaks in the EMW frequency range of 10 GHz to 18 GHz, which is caused by the two slow peaks of the *ε*″ curves in the same EMW frequency range.

It is worth noting that the *tan δ_ε_* curves (Figure 9c) of the mortar samples with W/C of 0.4 and S/C of 1 have a large shift compared with other samples in the EMW frequency range of 2 GHz to 18 GHz. This may be because the addition of quartz sand makes the *ε*″ value of the samples increase, but the *ε*′ value basically does not change, resulting in a significant shift in the *tan δ_ε_* curves. This experimental phenomenon shows that in the mortar samples with the S/C of 0 and 1, the dielectric loss effect is more substantial. That is, the EMW loss through the medium is greater than that of the mortar samples with S/C of 2 and 3.

The dielectric loss in cement-based materials primarily results from electromagnetic losses due to polarization (polarization loss). In the absence of an external electromagnetic field, the dipoles on the sample surfaces are randomly oriented. When exposed to an electromagnetic field, these dipoles continuously realign as the field’s direction changes, leading to the absorption of electromagnetic waves. Cement hydrates contain defect sites and isolated chemical groups, for example, irremovable intermediate active hydroxyl groups on the surface of calcium silicate hydrate gel (C-S-H) [54], which can play roles in permanent dipole polarization centers with inherent dipole moments [55]. The presence of cement hydrates gives cement-based materials a specific dielectric loss capacity.

In Figure 9b, the *μ*′ value of mortar test blocks with different cement–sand ratios fluctuates up and down from 1.1, while the *μ*″ value is mostly close to 0, which indicates that the tested mortar samples are basically non-magnetic. That is, under the action of an electromagnetic field, the electromagnetic loss caused by the rearrangement of the electric dipole moment in mortar samples is minimal, so the magnetic properties of the sample are very weak, and the magnetic loss capacity is extremely low. The *tan δ_μ_* values (Figure 9d) of mortar samples fluctuate randomly and irregularly with the EMW frequency, indicating that the sample has basically no eddy current loss and magnetic loss ability. It is worth noting that a large peak appears in the *tan δ_μ_* curve of the mortar sample with S/C of 2, which the metal oxides in cementitious materials may cause.

**Figure 10:**
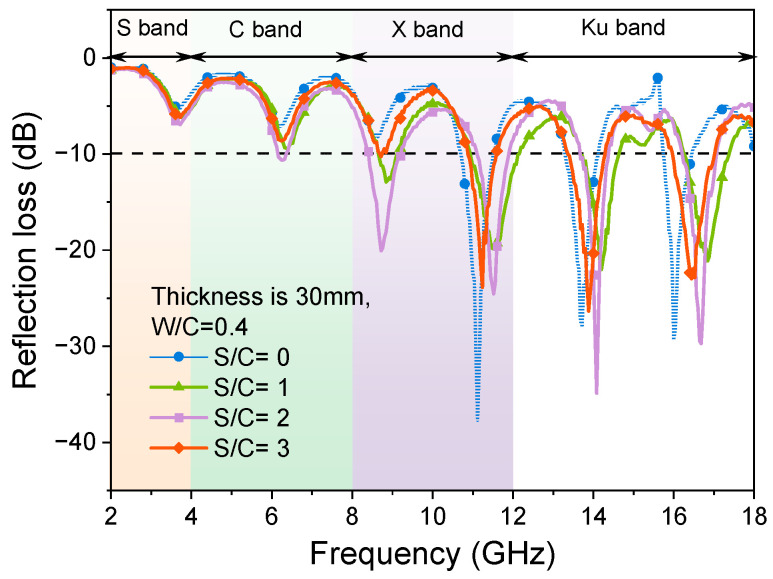
Theoretically calculated RL curves of samples with different S/C ratios, the same W/C of 0.4, and the same thickness of 30 mm.

Figure 10 shows the theoretically calculated EMW RL curves of samples with different S/C ratios, the same thickness of 30 mm, and the same W/C ratio of 0.4. Table 4 shows the effective absorption bandwidth of the theoretical calculation of the electromagnetic RL curve. In Figure 10, with the increase in quartz sand content, the EMW RL curves of the test samples move first downward and then upward, indicating that the addition of quartz sand improves the EMW-absorption capacity of the hardened mortar. In the middle and low-frequency EMW bands with a frequency of 2 GHz to 8 GHz, the RL curves of the samples basically coincide without obvious deviation, indicating that the change in electromagnetic parameters of the mortar by changing the quartz sand contents has little influence on the absorption of EMWs. In the range of EMW frequency from 8 GHz to 18 GHz, the RL curves of the samples show a trend to the left. According to the theoretical calculation of EMW RL curves, all the tested cement-based samples have poor absorption effects on EMWs. As shown in Table 4, mortar samples show slight absorption properties only in the high-frequency band. The maximum effective absorption bandwidth value is smaller than 4.6 GHz, but the RL value of the mortar sample can be as low as −37.69 dB.

#### 3.2.2. Samples with Different Water-to-Cement Ratios

**Figure 11:**
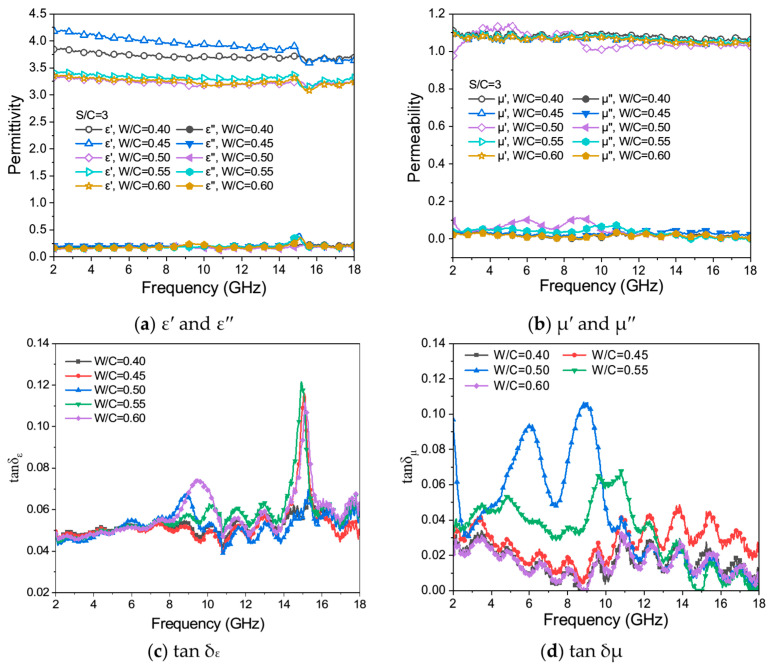
*ε*′ and *ε*″ (**a**), *μ*′ and *μ*″ (**b**), *tan δ_ε_* (**c**), and *tan δ_μ_* (**d**) for samples with different W/C ratios and the same S/C of 3.

Figure 11a–d shows the change curves of *ε*′ and *ε*″ (Figure 11a), *μ*′ and *μ*″ (Figure 11b), *tan δ_ε_* (Figure 11c), and *tan δ_μ_* (Figure 11d) of samples with different W/C ratios in the range of EMW frequencies from 2 GHz to 18 GHz.

From Figure 11a, it can be seen that the change in W/C ratios influences the dielectric real part *ε*′ of cementitious materials. With the increase in the W/C ratios of the test samples, *ε*′ shows a trend of first increasing and then decreasing, which may be because of the change in cement hydration product contents, which affects the electromagnetic parameters of the mortar. When the W/C ratio is greater than 0.5, the dielectric real part *ε*′ of the mortar is favorable. The values of the dielectric real part *ε*′ fluctuate in the range of 3.2 to 4.2, and an inflexion point appears at the EMW frequency of 15 GHz. Although the values of the dielectric virtual part *ε*″ fluctuate near 0, and a slow peak appears at the EMW frequency of 15 GHz, indicating that the mortar samples have a specific polarization ability under the action of the magnetic field, the leading dielectric loss is limited. In the EMW frequency range of 2 GHz to 8 GHz, the dielectric loss angle *tan δ_ε_* curves (Figure 11c) of mortar samples are generally relatively gentle. When the W/C ratio is 0.6, the *tan δ_ε_* curve of the mortar sample has two apparent peaks in the EMW frequency range of 8 GHz to 18 GHz, while the curves of the mortar samples with W/C of 0.50 and 0.55 have only one peak.

It can be seen from Figure 11b that the *μ*′ value of mortar samples with different W/C ratios fluctuates above and below 1.1, and *μ*″ is close to 0, indicating that the mortar sample has very weak magnetism and extremely low magnetic loss capacity. The *tan δ_μ_* values fluctuate randomly and irregularly with the increase in EMW frequencies, which further indicates that the mortar sample basically has no eddy current loss and magnetic loss ability.

**Figure 12:**
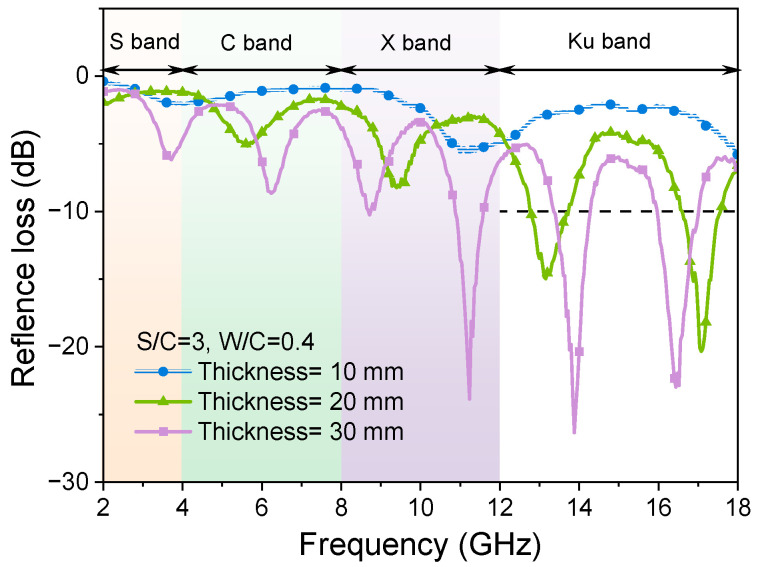
Theoretically calculated RL curves of mortar samples of different thicknesses and the same W/C of 0.4 and S/C of 3.

Figure 12 shows the theoretical curves of electromagnetic RL of mortar samples with different thicknesses when the W/C ratio is 0.4 and S/C is 3. When the thickness of the sample is 10 mm, the theoretically calculated curves show that the sample cannot effectively absorb EMWs. When the thickness increases to 20 mm and 30 mm, the absorption peaks of the RL curves of the samples move downward within the range of 2 GHz to 18 GHz. Meanwhile, the absorption effects of EMWs are gradually enhanced. At the same time, the absorption peak numbers increase while the primary absorption peaks shift to the low-frequency range. The quarter-wavelength theory can explain those phenomena [56,57,58], as shown in Equation (8).

(8) $$d=\frac{\left(2n+1\right)\lambda }{4}=\frac{\left(2n+1\right)c}{4f_{m}\sqrt{\varepsilon_{r}\mu_{r}}}\;\;\;\;\;\;\;\left(n=1,2,3\ldots \right)$$

where *d* represents the matching thickness of the mortar sample, λ represents the wavelength in the mortar sample, $f_{m}$ represents the matching frequencies of the absorbing peaks, c represents the light velocity, and *μ_r_* and *ε_r_* are the complex relative permeability and permittivity, respectively. The relationship between the absorption peak of mortar samples and their thicknesses accords with the theoretical equation. With the increase in the thickness of the sample, the frequency of the impedance-matching electromagnetic wave decreases, causing the reflection peak to shift to the low-frequency range of the electromagnetic wave. Therefore, the effective absorption band of cement-based electromagnetic wave-absorbing material can be adjusted by changing the thickness of the sample.

**Figure 13:**
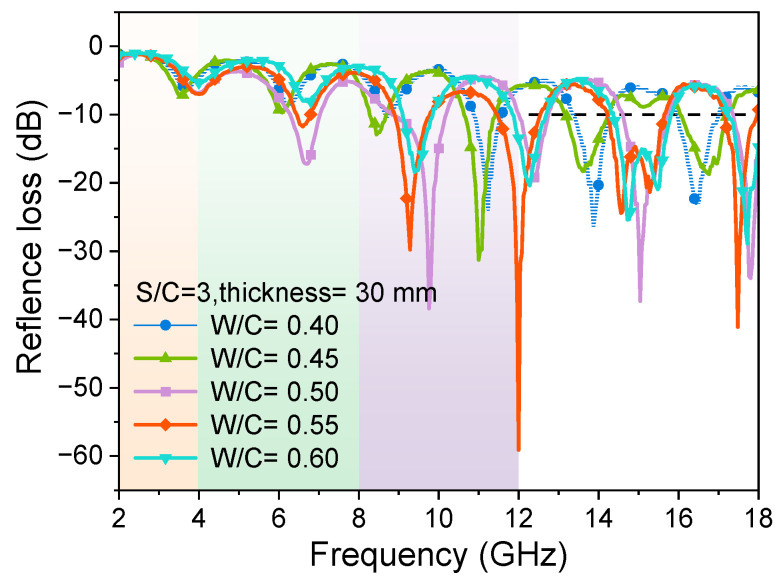
Theoretically calculated RL curves of mortar samples with different W/C ratios and the same S/C of 3 and thickness of 30 mm.

Figure 13 shows the theoretical curve of RL of mortar samples with different W/C ratios at a thickness of 30 mm in the EMW frequency range of 2 GHz to 18 GHz. In Figure 13, the RL curves of the samples in the middle- and high-frequency ranges of EMWs shift to the left, but their effective absorption bandwidth values are not ideal. The average effective absorption bandwidth of all samples is only 3.81 GHz, while the absorption of EMWs is mainly concentrated in the middle and high-frequency region of 8 GHz to 18 GHz. When the W/C of mortar is 0.55, the minimum absorption peak at the EMW frequency of 12 GHz can reach −59.12 dB.

## 4. Discussions

**Figure 14:**
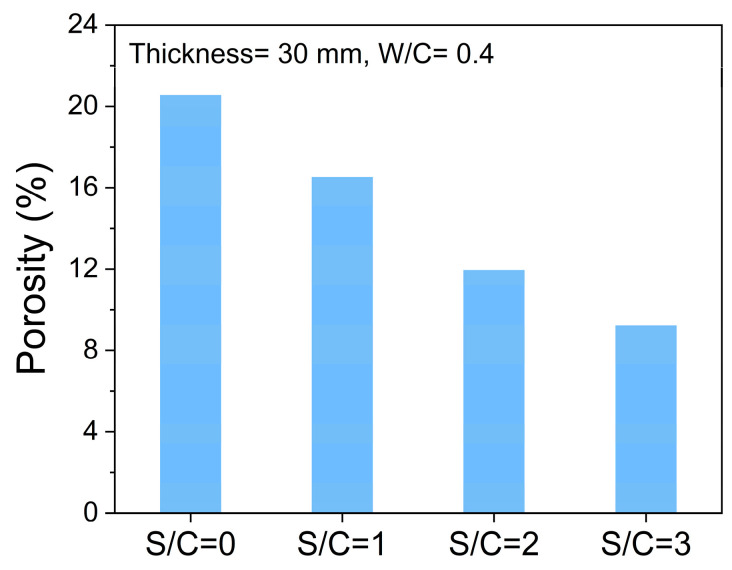
Oven-dry porosity values of samples with different S/C ratios and the same thickness of 30 mm and W/C of 0.4.

**Figure 15:**
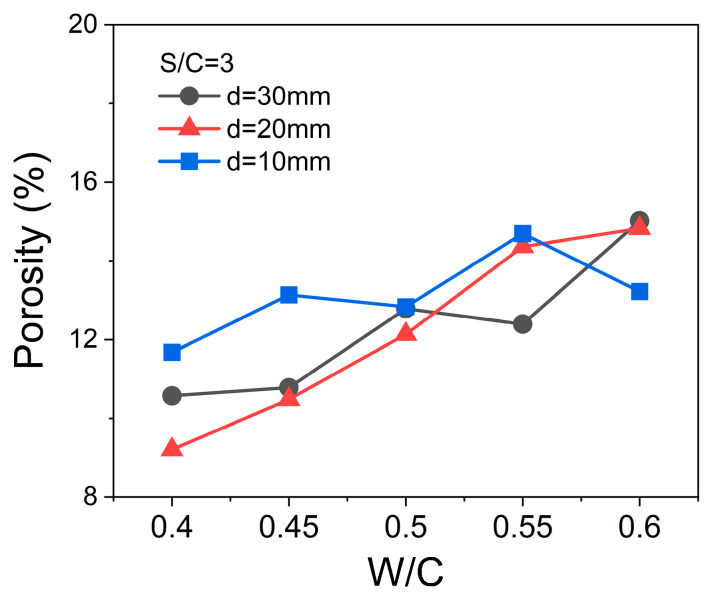
Oven-dry porosity values of samples with different W/C ratios and thicknesses, and the same S/C of 3.

By comparing the RL data in Section 3.1 and Section 3.2, for the mortar samples, there is a significant difference between the actual RL curves and the theoretical calculation results. The most significant difference between the calculated RL curves by the parameters obtained by the coaxial method and the measured RL curves by the bow-frame method is the roles of the porous structure and the air in the pores. According to Figure 14, the oven-dry porosity values of cement-based samples with a certain W/C ratio decrease with the increase in S/C ratios, proving that the open pore (containing air) volumes in the sample are affected by the contents of quartz sand. In Figure 15, it is easy to find that when the S/C ratio is constant, the oven-dry porosity values of the mortar samples increase with the increase in W/C ratios. Here, it should be noted that the thicknesses of mortar samples also influence the oven-dry porosities of the samples with the same S/C and W/C ratios. These may be the systematic errors caused by the molding of mortar samples. 

The influence of pore structure on the EMW-absorption performance of mortar is mainly reflected in the following aspects. Firstly, the interface between the air in the pores and the mortar matrix will cause EMW scattering and multiple reflections, increasing the propagation paths of EMWs in the mortar and enhancing the absorption performance of EMWs. Secondly, the pore sizes in the hardened mortar also affect the EMW-absorption performance of the sample. Pores of a specific size can resonate with EMWs at a specific frequency, thereby increasing the energy dissipation of EMWs. Larger pores can act as resonators, strongly absorbing EMWs of specific frequencies. Finally, the pore shape and volumes are also important factors affecting the EMW-absorption performance of the sample. Irregular pores can increase the scattering and reflection of EMWs, improving the EMW-absorption performance of mortar. Needle-like and elongated pores can guide EMWs in a specific direction inside the cementitious materials, increasing EMW reflectance loss [59]. Based on the similarity principle, the presence of air reduces the relative proportion of high dielectric constant components (such as cement hydrates) in the mortar and reduces the overall dielectric constant of the sample. 

**Figure 16:**
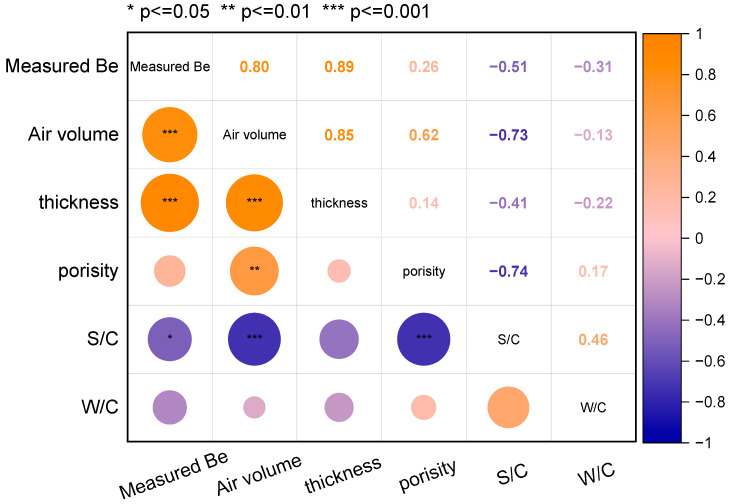
Correlation matrix among the measured effective bandwidth (B_e_), the actual air volume in the sample, thickness, porosity, S/C, and W/C of cement-based samples.

However, the EMW RL curves of the cement-based samples calculated from the electromagnetic parameters measured by the coaxial method assume that the tested powders are dispersed evenly in the paraffin (the tested samples are dense and uniform without pores). Thus, this work supposes that the actual measured electromagnetic RL curves of cementitious materials with sand are the comprehensive results of the porous structure and the electromagnetic parameters of the matrix. Figure 16 presents the Spearman’s correlation matrix image of the measured EMW effective bandwidth of cement-based specimens and the actual air volume in the samples, thickness, porosity, S/C, and W/C. Spearman’s correlation coefficient is a metric that quantifies the strength of a monotonic relationship between two ranked variables. Spearman’s correlation coefficients take a range of values from −1 to +1. For example, a value closer to +1 (the deep orange), as shown in the upper right triangle of the matrix Figure 16, indicates a strong monotonic positive correlation between those two variables in the horizontal axis and vertical axis. The asterisks in the subtriangular grids of Figure 16 indicate *p*-values from the statistical analysis, denoting the significance levels of the factors on the horizontal axis with respect to those on the vertical axis. One asterisk means the *p*-value is less than 0.05, two asterisks mean the *p*-value is less than 0.01. While three asterisk mean the *p*-value is less than 0.001. As to the meanings of colour in Figure 16, the shape of circles’ colour in the matrix represents the sizes of the correlation coefficient of the factors on the horizontal axis with respect to those on the vertical axis. In Figure 16, the change in Measured B_e_ positively linearly correlates to the actual air volume in the sample, the thickness of the sample, and the S/C ratio, where the significant level of the S/C ratio is smaller than that of the other two. This is because quartz sand, as a typical dielectric material, can reduce the dielectric constant of the cementitious material when added (Figure 9a), thus enhancing the impedance matching between the cementitious material and free space. The distribution of quartz sand in cementitious materials can also cause multiple reflections and scattering of EMWs, complicating the path of EMW propagation within the cementitious material. This increases the chances of EMWs interacting with the cementitious material, thus improving the EMW-absorption performance.

According to the previous analysis, the influences induced by the air in the porous structure of mortar cause the difference between the theoretically calculated and the measured EMW RL curves. Nevertheless, this difference also provides a reference for the design of cement-based EMW absorbers. In the field of building materials, it is often necessary to consider the balance between the cost, mechanical properties, durability, and EMW absorption properties. Porosity can be adjusted by controlling the preparation process of the mortar to obtain good electromagnetic absorption properties. However, it should be noted that excessive porosity may lead to a decline in the mechanical properties of the material [60]. The results of this study suggest that EMW-absorption properties of mortar can be controlled by changing the amount of quartz sand. The relatively low cost of quartz sand also provides strong support for its large-scale engineering practice.

Based on this, the mortar electromagnetic radiation-absorbing plate proposed in this study has broad application prospects in civil and military fields. It is suitable for buildings close to electromagnetic radiation sources, such as communication base stations, buildings near high-voltage lines, and places with special requirements for electromagnetic environments, such as precision medical equipment rooms and scientific research laboratories in hospitals. In addition, well-designed mortar slabs can be used to build storage, providing better EMW protection for weapons and equipment.

## 5. Conclusions

This work investigated the influences of quartz sand on the EMW-absorption performance of cementitious materials with different sand-to-cement (S/C) ratios, water-to-cement (W/C) ratios, and thicknesses of samples. The EMW RL curves were derived using the test measurement and theoretical calculations from the electromagnetic parameters of the sample powders. The following conclusions can be drawn:

1. The content variation of quartz sand in mortar samples with a constant W/C ratio significantly affects the EMW-absorption effective bandwidth values. The presence of quartz sand in the hardened cement-based materials regulates the oven-dry porosity values of samples, resulting in the change in air volumes in the matrix and the impedance-matching behaviours between cementitious materials and the EMW transparent phases (sand and air in the pores). The threshold of the S/C ratio of mortar is 3; that is, when the S/C ratio is larger than 3, the absorption of EMWs in the high-frequency band decreases sharply.
2. The thicknesses of the mortar with the same S/C ratio and W/C ratios significantly influence the absorption performance of EMWs because of the changing of actual air volumes in the mortar. It was found that a mortar sample with a thickness of 30 mm obtained good EMW absorption with a larger effective absorption bandwidth. This indicates that the optimization of sample thickness is an essential factor in improving the absorption performance when designing EMW-absorbing materials.
3. When the S/C ratio of a mortar sample is fixed, the change in W/C ratios will have little influence over the EMW-absorption performance of the mortar samples with the same thickness values. The variation in actual air volume values induced by higher W/C ratios in this work is not as much as that induced by the quartz sand contents in the mortar samples.
4. The air within the porous structure and the sand in the solid skeleton of the mortar samples influence the EMW effective bandwidth in a coordinated way. The calculated electromagnetic parameters of mortar powder samples can qualitatively describe the effects of solid matrix variation caused by S/C and W/C ratios on EMW-absorption performance. The effective bandwidth of EMW absorption of a mortar sample is positively linearly related with the S/C ratios and the thicknesses of the sample. Therefore, the calculated EMW RL curves can hardly precisely describe the real EMW-absorption performance of mortar samples.

## Figures and Tables

**Table 1:** The effective bandwidth B_e_ and minimum values in reflectance curves (RL_min_) of slab-like mortar samples in the literature.

Reference	W/C	S/C	Thicknesses (mm)	B_e_ (GHz)	RL_min_ (dB)
[13]	0.30	0.24	20	1.28	−19.47
[29]	0.33	1.5	20	2.31	−13.70
[30]	0.30	1.5	25	1.54	−14.47
[31]	0.50	3	10	-	−7.70
[32]	0.20	1	20	4.25	−23.07

**Table 2:** Chemical composition of silicate cement (wt.%).

**Components**	Na_2_O	MgO	Al_2_O_3_	SiO_2_	P_2_O_5_	SO_3_	Cl	K_2_O	CaO	TiO_2_	Fe_2_O_3_	Others
**Content**	0.80	4.52	5.31	21.01	0.17	3.67	0.15	1.18	58.90	0.28	3.81	0.22

**Table 3:** Mix proportions of cementitious samples.

Group	W/C	S/C	Thicknesses (mm)
Ref-0.40-10	0.4	3	10
Ref-0.40-20	0.4	3	20
Ref-0.40-30	0.4	3	30
Ref-0.45-10	0.45	3	10
Ref-0.45-20	0.45	3	20
Ref-0.45-30	0.45	3	30
Ref-0.50-10	0.5	3	10
Ref-0.50-20	0.5	3	20
Ref-0.50-30	0.5	3	30
Ref-0.55-10	0.55	3	10
Ref-0.55-20	0.55	3	20
Ref-0.55-30	0.55	3	30
Ref-0.60-10	0.6	3	10
Ref-0.60-20	0.6	3	20
Ref-0.60-30	0.6	3	30
Ref-0.40-30	0.4	3	30
Ref-0.40-30	0.4	2	30
Ref-0.40-30	0.4	1	30
Ref-0.40-30	0.4	0	30

**Table 4:** Theoretical calculations result in an effective absorption bandwidth and RL_min_.

Group	S/C	W/C	Thickness (mm)	B_e_ (GHz)	RL_min_ (dB)
Ref-0.40-0-30	0	0.40	30	2.35	−37.69
Ref-0.40-1-30	1	0.40	30	3.71	−22.04
Ref-0.40-2-30	2	0.40	30	3.35	−34.89
Ref-0.40-3-30	3	0.40	30	2.75	−26.34
Ref-0.45-3-30	3	0.45	30	3.47	−31.26
Ref-0.50-3-30	3	0.50	30	4.51	−38.38
Ref-0.55-3-30	3	0.55	30	4.55	−59.12
Ref-0.60-3-30	3	0.60	30	3.79	−28.92
Ref-0.40-3-10	3	0.40	10	0	−5.80
Ref-0.40-3-20	3	0.40	20	1.96	−20.34

## Data Availability

The original contributions presented in this study are included in the article. Further inquiries can be directed to the corresponding authors.
